# SRF ISOFORMS DIFFERENTIALLY IMPACT MITOCHONDRIAL FUNCTION AND AGING

**DOI:** 10.1093/geroni/igae098.2302

**Published:** 2024-12-31

**Authors:** Shakshi Sharma, Xiaomin Zhang, Gohar Azhar, Jeanne Y Wei

**Affiliations:** University of Arkansas for Medical Sciences, LITTLE ROCK, Arkansas, United States; University of Arkansas for Medical Sciences, Little Rock, Arkansas, United States; University of Arkansas for Medical Sciences, Little Rock, Arkansas, United States; University of Arkansas for Medical Sciences, Little Rock, Arkansas, United States

## Abstract

Serum response factor (SRF) is a highly conserved widely expressed transcription factor that plays a key role in regulating a number of muscle specific genes in various tissues, including cardiac and skeletal muscles. Alternative splicing (AS) of SRF generates multiple mRNA transcripts and protein isoforms. The SRF isoforms are critical in regulation of SRF target genes and increases in the human heart with advancing age. We cloned novel isoforms SRF-Δ3 and SRF- Δ345 from human tissues and studied their effect on mitochondrial function. Three SRF isoforms (Wild Type, Δ-3, Δ-345) were subcloned into expression vectors. Muscle cell line C2C12 was transfected with SRF-empty vector, SRF-WT, SRF-Δ3, and SRF-Δ345, respectively. Gene expression was determined by RT-PCR and Western blotting. The mitochondrial function was determined by the SeahorseXFe96 analyzer. SRF-Δ3 and SRF- Δ345 significantly reduced the oxygen consumption rate (OCR) and glycolysis (ECAR) in C2C12 cells as compared to SRF-WT. The protein and gene expression of PGC-1α, PGC-1β, MFN 1, MFN 2, Fis1 and Opa1 were also differentially impacted by SRF isoforms. Further, the SRF isoforms significantly impact the protein expression of mitochondrial electron chain complex OXPHOS (I-IV) proteins, thus affecting mitochondrial function. These findings showed the potential effects of the SRF isoforms on mitochondrial function, which might affect cellular senescence and aging.

